# SUICIDE RISK ASSESSMENT AND MANAGEMENT IN THE CONTEXT OF TRANSITION TO LONG-TERM CARE

**DOI:** 10.1093/geroni/igz038.3040

**Published:** 2019-11-08

**Authors:** Douglas W Lane

**Affiliations:** VA Puget Sound Healthcare System, Geriatrics and Extended Care Svc, Tacoma, Washington, United States

## Abstract

The transition from independent living to living in a supported care facility can create significant emotional stress for the older person undergoing the move. From the perspective of suicide risk management, it is vital to appreciate the potential increase in risk associated with this period of adjustment. In this case-based talk, we will discuss key issues in assessing and managing suicide risk in this context. Factors in risk management to be discussed include the person’s social/family context, baseline psychological status, coping assets, and intraindividual values and diversity characteristics. Emphasis will be placed on the need for active assessment, mobilization of resiliency factors, and monitoring during the phase of adjustment. The need to ensure coordination of care across settings will be discussed as well. Finally, we will identify the ways in which the transition can be undermined by ageism on the part of professionals and/or the person’s loved ones.

